# Impact of BMI and PRP Platelet and Red Blood Cell Content on the Coagulation Kinetics of Ortho-R/PRP Mixtures

**DOI:** 10.3390/polym17111515

**Published:** 2025-05-29

**Authors:** Anik Chevrier, Marc Lavertu

**Affiliations:** 1 Department of Chemical Engineering, Polytechnique Montreal, 2500 Chem. de Polytechnique, Montreal, QC H3T 1J4, Canada; a.chevrier@polymtl.ca; 2 Institute of Biomedical Engineering, Polytechnique Montreal, 2500 Chem. de Polytechnique, Montreal, QC H3T 1J4, Canada

**Keywords:** chitosan, platelet-rich plasma, thromboelastography

## Abstract

Ortho-R (ChitogenX Inc., Kirkland, QC, Canada) is a lyophilized chitosan formulation that also contains calcium chloride and trehalose. Ortho-R was designed to be solubilized in autologous platelet-rich plasma (PRP), a blood-derived component, in order to become an injectable implant that augments the surgical repair of soft tissues. The Ortho-R/PRP formulation coagulates post-application, similarly to blood. Having the ability to predict the speed of coagulation of an Ortho-R/PRP mixture prepared with PRP isolated from a specific patient would be an advantage in the operating room. The purpose of this study was to investigate whether human donor characteristics (age, sex, body mass index, habits) and autologous PRP properties would have an impact on Ortho-R/PRP mixture coagulation. Clot maximal amplitude and shear elastic modulus were significantly positively correlated with body mass index and platelet concentration in the isolated PRPs. Clot formation time, maximal amplitude and shear elastic modulus were all negatively correlated with PRP red blood cell concentration (and associated hemoglobin and hematocrit content). Donor characteristics were not good predictors of coagulation kinetics in Ortho-R/PRP mixtures. Some of the isolated PRP properties were better predictors of Ortho-R/PRP coagulation kinetics. However, predicting how an Ortho-R/PRP mixture from a particular patient will coagulate is very difficult since all PRP isolation devices yield heterogeneous PRPs and analysis of the isolated PRPs occurs post-administration.

## 1. Introduction

Ortho-R (ChitogenX Inc., Kirkland, QC, Canada) is a lyophilized finished product that contains chitosan, a polymer derived from chitin, as well as calcium chloride and trehalose [1,2]. The product was designed to be soluble in autologous platelet-rich plasma (PRP), a biologic that is obtained through centrifugation of anti-coagulated whole blood. The Ortho-R product has been used to improve soft tissue repair in many orthopedic pre-clinical models [3,4,5,6,7]. Clinically, Ortho-R was used in a randomized controlled trial as an adjunct to improve the surgical repair of torn rotator cuff tendons.

Ortho-R/PRP mixtures are injectable, and they coagulate in a manner similar to that of clotting blood post-injection [8], through the action of calcium chloride on the PRP portion of the mixture, and coagulation kinetics can be assessed with thromboelastography [9,10]. We found during the clinical trial that the coagulation of Ortho-R/PRP was slower when human PRP was used instead of animal PRP. Therefore, the solubilized Ortho-R/PRP mixture was pre-incubated for 30–45 min until it became viscous and adhesive prior to arthroscopic delivery to the rotator cuff. It would be advantageous to identify the variables that influence the coagulation kinetics of the Ortho-R/PRP mixtures (for example, the age or sex of the patients) in order to know at what time the product should be solubilized in PRP during surgery in order to ensure that the properties of the Ortho-R product are optimal at the time of its arthroscopic delivery.

A literature review on the coagulation kinetics of blood allowed us to identify several variables that are likely to influence the coagulation of the Ortho-R product since it is prepared with PRP, a blood derivative. Hypercoagulability was reported in older subjects compared to younger subjects [11,12,13,14,15], in female subjects compared to male subjects [12,13,15,16,17,18,19,20,21,22], in subjects who are healthy but obese compared to age- and sex-matched healthy individuals of normal weight [23], in trauma patients who are obese [24] and in female subjects taking oral contraceptives [12,25,26,27,28]. Hypocoagulability has been reported in subjects taking small doses of aspirin as a preventive measure [19,29], in smokers compared to non-smokers [30] and in male subjects who had consumed alcohol [31]. Bleeding abnormalities and an increased risk of venous thromboembolism have been reported in patients using antidepressants [32,33]. In addition, platelet counts in blood have been shown to strongly influence many of the coagulation kinetics parameters measured by thromboelastography [34,35,36].

Our starting hypothesis for this project was that Ortho-R/PRP mixtures would coagulate more quickly when PRP isolated from older and heavier individuals and females was used for product solubilization compared to younger and leaner individuals and males. In addition, we hypothesized that platelet content in PRP would significantly affect the coagulation kinetics of the PRP mixtures, with higher platelet concentrations leading to quicker coagulation and yielding stiffer hybrid chitosan–PRP clots. We also included a questionnaire in our study to control for the following categorical variables: the consumption of aspirin, oral contraceptives, alcohol and antidepressants, and smoking.

## 2. Materials and Methods

### 2.1. Preparation of the Ortho-R Freeze-Dried Formulation

Chitosan powder with a number-average molar mass (*M*_n_) of 41 kDa, polydispersity index (PDI) of 1.8 and degree of deacetylation (DDA) of 83.4% (produced in-house) was hydrated in sterile water for injection (Product N° L8500, B Braun, Melsungen, Germany) for 24 h. Hydrochloric acid (1N) was then added (Product N° 1.09057.1000, Merck KGaA, Darmstadt, Germany), and the solution was stirred for 24 h to dissolve the chitosan. Stock calcium chloride (Product N° CA140-500G, Spectrum, New Brunswick, NJ, USA) and trehalose (Product N° T-104-4, Pfanstiehl, Waukegan, IL, USA) solutions were then added to the chitosan solution. The final chitosan formulation contained 1% (*w*/*v*) chitosan, 29 mM hydrochloric acid, 42 mM calcium chloride (for PRP solidification) and 1% (*w*/*v*) trehalose (as a lyoprotectant). The chitosan solution was aliquoted in individual vials (5 mL into 10 mL glass vials) for freeze-drying. The 55 h freeze-drying cycle consisted of the following: (1) several-step cooling and isothermal steps until freezing at −35 °C; (2) ramp to −7 °C, then isothermal for 1925 min; (3) ramp to 40 °C, then isothermal for 360 min; (4) ramp to 20 °C, then isothermal for 6 h, all at chamber pressure (Pc) = 67 mTorr. A single lot of Ortho-R was used throughout the study. The reconstitution time of a vial in water was 3 min, and the resulting solution had a pH of 6.183 and an osmolality of 163 mOsm/kg.

### 2.2. Donor Recruitment

The study was approved by the Institutional Review Board of Polytechnique Montreal (protocol CER-2223-24-D, approved 29 September 2022). Sixty healthy donors were recruited via web and bill posting, and all signed an informed consent form. The donor characteristics can be found in Table 1.

Inclusion criteria:

- Donors were healthy (state of complete physical, mental and social well-being);
- Donors felt well on the day of the blood draw;
- Donors were not fasting on the day of the blood draw;
- Donors had not given blood for 56 days (men) or 84 days (women);
- Donors were aged between 18 and 75 years;
- Donors were able give their free and informed consent.

Exclusion criteria:

- Donors had a fever > 37.5 °C;
- Donors had previously been diagnosed with a coagulopathy (e.g., hemophilia, Von Willebrand disease, clotting factor deficiencies) or blood abnormalities (e.g., low hemoglobin);
- Donors had received a vaccine and/or had had surgery in the last 56 days;
- Donors were pregnant or were nursing and had given birth less than 6 months before;
- Donors were taking medications that affect blood clotting (except low-dose aspirin), including warfarin, frusemide, penicillin and ranitidine;
- Donors had infectious diseases, HIV/AIDS, sepsis and/or hepatitis A, B, C.

### 2.3. Blood Draw and Isolation of PRP

A nurse collected the blood samples by arm venipuncture using 21 g Vacutainer butterfly sets (Product N° 367281, BD, Franklin Lakes, NJ, USA). The sequence for blood collection was as follows: (1) one 3 mL Vacutainer EDTA tube (Product N° 367841, BD, Franklin Lakes, NJ, USA) for a complete blood count and platelet count (DxH520 hematology analyzer, Beckman, Brea, CA, USA); (2) 52 mL of blood anti-coagulated with 8 mL of acid citrate dextrose-A (ACD-A) for PRP isolation. A uniform device was used to prepare the PRP. The ACD-A, centrifuge and all the materials used to isolate the PRP were kindly provided by Zimmer Biomet (Warsaw, IN, USA). The 60 mL of ACD-A anti-coagulated blood was loaded into the GPS^®^ III PRP isolation device (Product N° 800-1004A, Zimmer Biomet, Warsaw, IN, USA) and centrifuged at 3200 rpm for 15 min. At the end of the isolation procedure, ~6 mL of PRP was collected from each device. The isolated PRP was also subjected to a complete blood count and platelet count (DxH520 hematology analyzer, Beckman, Brea, CA, USA).

### 2.4. Thromboelastography

An isovolume of PRP (5 mL) was injected into the Ortho-R vial, and the vial was shaken vigorously for 20 s. Thromboelastography was performed immediately using a Haemoscope TEG 5000 instrument (Haemonetics, Boston, MA, USA) to map the coagulation kinetics of the Ortho-R/PRP mixtures. 360 µL of Ortho-R/PRP mixture was loaded into a thromboelastography cup pre-warmed at 37 °C, and the assay was run until maximal amplitude was reached. The parameters measured included the time of initiation for clotting (R = Clot reaction time), how quickly the clot forms (k = Clot formation time and α angle), the final strength of the clot (MA = Maximal amplitude and G = Shear elastic modulus) and the time it takes to reach maximum clot strength (TMA = Time to maximal amplitude).

### 2.5. Statistical Analysis

All statistical analyses were performed with SAS Enterprise Guide 7.1 and SAS 9.4 or with GraphPad Prism 10. Data in the results section are presented as average ± standard deviation (AVE ± SD). The *t*-test task in SAS Enterprise Guide was used to compare the different groups. Correlations between continuous variables were analyzed by calculating the Pearson correlation coefficients (r). Point biserial correlations between continuous variables and dichotomous categorical variables (sex and oral contraceptive consumption for females) were calculated. *p* values of *p* < 0.05 were considered significant.

## 3. Results

### 3.1. Donors

In total, 27 male and 33 female healthy donors were recruited in Montréal, QC, Canada, for the study (Table 1). There was no difference between male and female donors in terms of age and body mass index (*p* = 0.6742 and *p* = 0.1387, respectively). None of the donors were taking low-dose aspirin, had consumed alcohol that day or were diabetic. Ten female donors were taking oral contraceptives, five donors were smokers (two males and three females) and two donors were taking antidepressants (one male and one female).

### 3.2. PRP Isolation

The partial complete blood count results from whole blood are shown in Table 2, and the complete dataset can be found in Supplementary Table S1. The values were all within normal reference ranges. Most of the parameters measured were similar in males and females. However, females had higher platelet counts than males (*p* = 0.0006), and males had higher red blood cell content (*p* < 0.0001), hemoglobin content (*p* < 0.0001), hematocrit content (*p* < 0.0001) and mean corpuscular hemoglobin concentration (*p* < 0.0001) than females.

The partial complete blood count results from isolated PRP are shown in Table 3, and the complete dataset can be found in Supplementary Table S2. There was great variation between the PRPs isolated from the different donors, as revealed by the large standard deviations. On average, isolated PRP had 1451 ± 553 × 10^9^/L platelets (5.7× that of whole blood, *p* < 0.0001), 23.4 ± 9.5 × 10^9^/L white blood cells (3.7× that of whole blood, *p* < 0.0001), 1.4 ± 1.1 × 10^12^/L red blood cells (0.3× that of whole blood, *p* < 0.0001), 40.8 ± 32.4 g/L hemoglobin (0.3× that of whole blood, *p* < 0.0001) and 0.13 ± 0.10 L/L hematocrit (0.3× that of whole blood, *p* < 0.0001). Almost all the parameters measured were similar in males and females. However, females had higher % lymphocytes than males (*p* = 0.0358), and males had a higher mean corpuscular hemoglobin concentration (*p* = 0.0186) than females (Supplementary Table S2).

### 3.3. TEG Data

**Figure 1:**
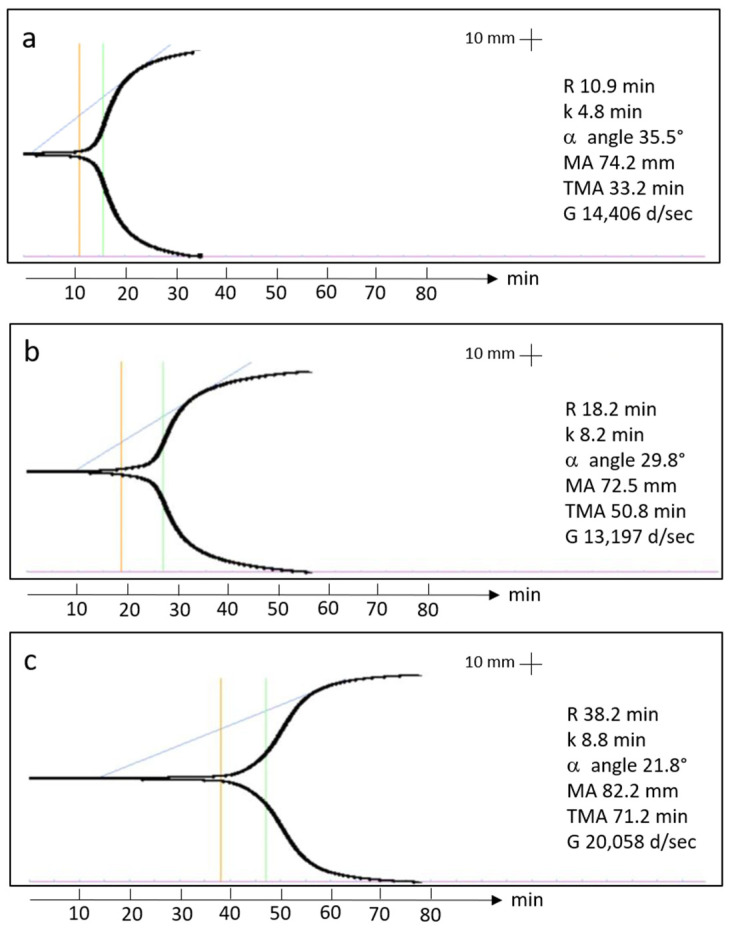
TEG tracings from two 20-year-old females (**a**,**b**) and one 20-year-old male (**c**) showing the clot reaction time (orange line), the clot formation time (green line) and the α angle (blue line).

Examples of thromboelastography tracings can be found in Figure 1. The coagulation kinetics data of the Ortho-R/PRP mixtures are shown in Table 4. There was great variability between donors for all the parameters tested. For example, clot reaction time, which corresponds to the time between assay initiation and when the thromboelastic traces have diverged by 2 mm, ranged from 10.4 min to 38.2 min. Similarly, the maximal amplitude, which corresponds to clot strength, ranged from 61.1 mm to 82.7 mm. There was no difference between males and females in terms of clot reaction time (*p* = 0.6033), clot formation time (*p* = 0.2030), α angle (*p* = 0.3371), maximal amplitude (*p* = 0.3173), time to maximal amplitude (*p* = 0.2835) or shear elastic modulus (*p* = 0.3419).

### 3.4. Correlations

**Figure 2:**
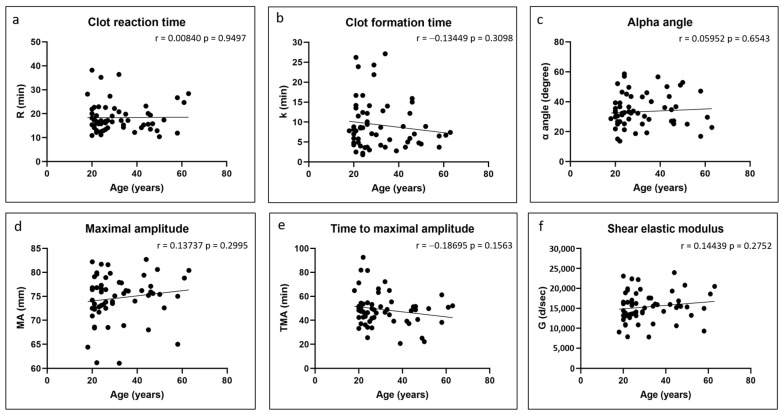
Linear regression plots showing Pearson correlation coefficients r and corresponding *p* values between TEG data and donor age.

**Figure 3:**
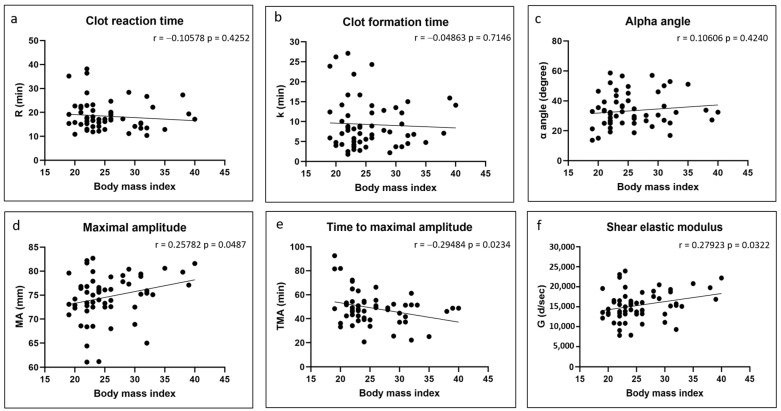
Linear regression plots showing Pearson correlation coefficients r and corresponding *p* values between TEG data and donor body mass index.

**Figure 4:**
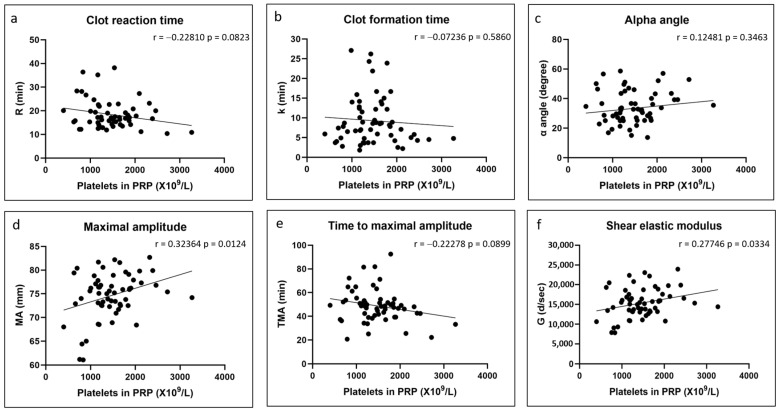
Linear regression plots showing Pearson correlation coefficients r and corresponding *p* values between TEG data and PRP platelet content.

**Figure 5:**
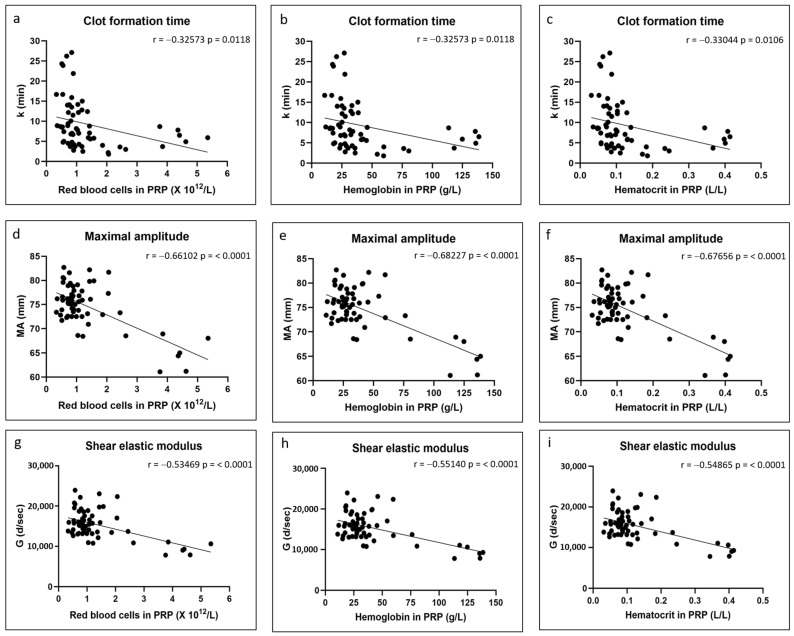
Linear regression plots showing Pearson correlation coefficients r and corresponding *p* values between TEG data and PRP red blood cell content (**a**,**d**,**g**), PRP hemoglobin content (**b**,**e**,**h**) and PRP hematocrit content (**c**,**f**,**i**).

There were no significant correlations between any of the coagulation kinetics parameters and donor age (Figure 2). Weak but significant positive correlations were found between maximal amplitude and shear elastic modulus and donor body mass index, while a weak but significant negative correlation was found between time to maximal amplitude and body mass index (Figure 3). Significant positive correlations were found between maximal amplitude and shear elastic modulus and PRP platelet concentration (Figure 4). Weak but significant negative correlations were found between clot formation time and PRP red blood cell, hemoglobin and hematocrit concentrations (Figure 5). Moderate and significant negative correlations were found between maximal amplitude and shear elastic modulus and PRP red blood cell, hemoglobin and hematocrit concentrations (Figure 5). There were no significant correlations between any coagulation kinetics parameters and sex (males versus females) or oral contraceptive consumption for females. We did not calculate biserial correlations for the other categorical variables (aspirin, alcohol and antidepressant consumption, and smoking and diabetes) due to their absence and/or low numbers (n = 0–5 out of a total n = 60).

## 4. Discussion

Our starting hypothesis that PRP isolated from females and from older and heavier individuals would lead to faster coagulation of Ortho-R/PRP mixtures was rejected. We found no difference between clot reaction time, clot formation time and α angle in female versus male donors, and no correlation was found between those three coagulation kinetics parameters and donor age and body mass index. Similarly to others [23,24], we found that the clot maximal amplitude and the shear elastic modulus were higher in the heavier donors. The time to reach maximal amplitude was also shorter in those individuals. It is possible that our cohort was too small and that our ranges of age and body mass index were too narrow to detect subtle differences in some of the coagulation kinetics parameters. However, it is more likely that previously published studies that reported on the effects of sex [12,13,15,16,17,18,19,20,21], age [11,12,13,14,15] and body mass index [23,24] on coagulation kinetics are not applicable to our study since they used whole blood and we used Ortho-R solubilized in PRP, which has very different properties from whole blood, as revealed by our complete blood count data.

Our male and female donor cohorts were well matched and had similar ages, body mass index and complete blood count data in general. Like others [12,22], we found that our female donors had higher blood platelet counts than male donors and that our male donors had higher hemoglobin and hematocrit concentrations than our female donors. One major criticism directed toward all PRP isolation devices is that they yield PRPs that are heterogeneous and that it is impossible to predict the concentrations of the different blood components in the isolated PRPs based on the starting material, whole blood, which is what we saw here. However, although there was great variability in the properties of the isolated PRPs, on average, they had significantly higher platelet and white blood cell counts and lower red blood cell counts (and associated hemoglobin and hematocrit) than whole blood. We also found great variability in the Ortho-R/PRP mixture coagulation kinetics. The Ortho-R/PRP mixtures were slower to coagulate (R = 10.4–38.2 min; k = 1.8–27.1 min; α angle = 13.7–58.6°) than what has been reported for anti-coagulated whole blood in healthy donors (R = 2.7–10.6 min; k = 0.7–3.6 min; α angle 44.9–77.7°) [16,37,38]. We believe that there are two reasons for this: (1) Previous studies reported coagulation kinetics parameters that were acquired by activating citrated whole blood with kaolin. Kaolin is a negatively charged fine clay that activates viscoelastic tests by contact (or intrinsic pathway) activation [39]. (2) The viscosity of the Ortho-R/PRP mixtures may slow down enzymatic processes. We have recently observed similar effects when using fibrin glues with chitosan solutions of different viscosities (unpublished observations).

Our data partially supported our second hypothesis that Ortho-R/PRP mixtures prepared with PRPs with higher platelet concentrations would coagulate quicker and yield stiffer hybrid chitosan–PRP clots. The hybrid chitosan–PRP clots prepared with PRPs with higher platelet concentrations were indeed stiffer, as revealed by an increase in maximal amplitude and in shear elastic modulus. This was also previously seen by others when using whole blood with different platelet concentrations [34,35,36]. Since the primary contributors to maximal amplitude are platelets and fibrin, and since shear elastic modulus is calculated based on the amplitude value until the maximum amplitude is reached, it is not surprising that the strength of hybrid chitosan–PRP clots prepared with higher platelet concentrations would be higher. To our surprise, and in contrast to previous studies [34,35,36,40], we found no effect of platelet concentration on the coagulation kinetics parameters that assess the speed of coagulation (clot reaction time, clot formation and α angle). We suspect that this is because our isolated PRPs were highly concentrated in platelets (1451 ± 553 × 10^9^/L) compared to the blood used in previous studies (<~350 × 10^9^/L). Thrombin generation occurs in stages (initiation, amplification and propagation) at the surface of platelets. It is possible that enough thrombin was generated at the platelet concentrations we tested to cleave all the available fibrinogen so that further increasing the platelet concentration would have no effect on the speed of coagulation.

The influence of red blood cell content on hybrid clot formation time, maximal amplitude and shear elastic modulus was an unexpected finding initially. We have since found manuscripts that showed that blood with increased hematocrit or hemoglobin had reduced clot strength as assessed by thromboelastography [40,41,42,43]. We hypothesize that this may be related to the platelet-mediated clot contraction that occurs following coagulation, especially at the later stages of clot formation, and that plays a major role in clot stiffening [44,45]. Activated platelets adhere to fibrin fibers through GPIIb-IIIa receptors and apply mechanical forces to the fibrin network, which leads to clot contraction and serum expulsion. Clots will undergo clot contraction, which increases stiffness and maximal amplitudes and correlates with platelet concentration, as shown by us here and by others [34,35,36]. The red blood cells present in the clots cannot be compressed and, therefore, a high content of red blood cells in the chitosan–PRP clots would be expected to impede clot contraction and thus lead to decreased maximal amplitude and shear elastic modulus. We were also initially surprised that increased red blood cell content in PRP (and the associated hemoglobin and hematocrit) led to a decrease in clot formation time. Clot formation time, along with α angle, measures the amplification and propagation phases of thrombin generation. Since then, we have found that some authors have shown that a subset of red blood cells express phosphatidylserine on their surfaces and that they directly contribute to thrombin generation [46,47].

As mentioned above, one limitation of this study is the low number of participants and the narrow ranges in age and body mass index. Another limitation is that we did not quantify fibrinogen in our PRP samples. We would expect the fibrinogen concentration in PRP to strongly influence Ortho-R/PRP coagulation [48,49,50]. It would also have been interesting to find out if the inflammatory cytokines present in isolated PRPs affect the coagulation kinetics of Ortho-R/PRP mixtures, as inflammation is a known initiator of coagulation [51].

To summarize, donor characteristics (age, body mass index and sex) were poor predictors of the coagulation kinetics of Ortho-R/PRP mixtures. Some of the coagulation kinetics parameters (clot formation time, maximal amplitude and shear elastic modulus) were weakly or moderately correlated with some PRP properties (platelet, red blood cell, hemoglobin and hematocrit concentrations). In view of the variability of the PRP obtained from the isolation devices and since analysis of PRP complete blood counts prior to surgical application seems unrealistic, we are unable to issue patient-specific recommendations on the pre-incubation time of the Ortho-R/PRP mixtures. However, based on what was observed during the clinical trial, we would maintain the 30-45 min pre-incubation time prior to application.

## Figures and Tables

**Table 1:** Donor characteristics.

Donor	Age (years) AVE ± SD (Min–Max)	BMI AVE ± SD (Min–Max)	Low-Dose Aspirin	Oral Contraceptives	Smoking	Alcohol That Day	Diabetes	Antidepressants
All (n = 60)	31 ± 12 (18–63)	25 ± 5 (19–40)	n = 0	n = 10	n = 5	n = 0	n = 0	n = 2
Males (n = 27)	31 ± 12 (20–61)	24 ± 4 (21–39)	n = 0	N/A	n = 2	n = 0	n = 0	n = 1
Females (n = 33)	32 ± 13 (18–63)	26 ± 6 (19–40)	n = 0	n = 10	n = 3	n = 0	n = 0	n = 1

BMI: Body mass index; N/A: Not applicable.

**Table 2:** Partial complete blood count data from whole blood.

Donors	Platelets (×10^9^/L) AVE ± SD	WBC (×10^9^/L) AVE ± SD	RBC (×10^12^/L) AVE ± SD	HB (g/L) AVE ± SD	HT (L/L) AVE ± SD
All (n = 60)	253 ± 74	6.4 ± 1.8	4.8 ± 0.4	146.2 ± 12.9	0.43 ± 0.03
Males (n = 27)	219 ± 45	6.1 ± 1.8	5.1 ± 0.3	157.4 ± 6.3	0.45 ± 0.02
Females (n = 33)	281 ± 82	6.6 ± 1.8	4.5 ± 0.3	137.1 ± 9.0	0.40 ± 0.02

WBC: White blood cells; RBC: Red blood cells; HB: Hemoglobin; HT: Hematocrit.

**Table 3:** Partial complete blood count data from isolated PRP.

Donors	Platelets (×10^9^/L) AVE ± SD	WBC (×10^9^/L) AVE ± SD	RBC (×10^12^/L) AVE ± SD	HB (g/L) AVE ± SD	HT (L/L) AVE ± SD
All (n = 60)	1451 ± 553	23.4 ± 9.5	1.4 ± 1.1	40.8 ± 32.4	0.13 ± 0.10
Males (n = 27)	1339 ± 435	22.5 ± 8.3	1.2 ± 1.0	38.0 ± 29.8	0.12 ± 0.09
Females (n = 33)	1542 ± 625	24.2 ± 10.4	1.4 ± 1.2	43.1 ± 34.7	0.13 ± 0.11

WBC: White blood cells; RBC: Red blood cells; HB: Hemoglobin; HT: Hematocrit.

**Table 4:** TEG data from Ortho-/PRP mixtures.

Donors	R (min) AVE ± SD (Min–Max)	k (min) AVE ± SD (Min–Max)	α Angle (°) AVE ± SD (Min–Max)	MA (mm) AVE ± SD (Min–Max)	TMA (min) AVE ± SD (Min–Max)	G (dyn/s) AVE ± SD (Min–Max)
All (n = 60)	18.4 ± 6.1 (10.4–38.2)	9.3 ± 6.1 (1.8–27.1)	33.3 ± 12.9 (13.7–58.6)	74.6 ± 4.8 (61.1–82.7)	48.9 ± 13.7 (20.7–92.5)	15,377 ± 3640 (6849–23,993)
Males (n = 27)	18.9 ± 6.5 (12.2–38.2)	10.4 ± 6.2 (2.5–27.1)	31.6 ± 9.7 (18.6–56.6)	73.9 ± 5.2 (61.1–82.7)	50.9 ± 10.4 (20.7–72.2)	14,862 ± 3757 (7849–23,933)
Females (n = 33)	18.0 ± 5.8 (10.4–35.2)	8.4 ± 6.0 (1.8–26.2)	34.7 ± 14.9 (13.7–58.6)	75.2 ± 4.4 (64.4–81.7)	47.2 ± 15.8 (22.2–92.5)	15,784 ± 3549 (9051–22,374)

R: Clot reaction time; k: Clot formation time; MA: Maximal amplitude; TMA: Time to maximal amplitude; G: Shear elastic modulus.

## Data Availability

The original contributions presented in the study are included in the article/Supplementary Material, further inquiries can be directed to the corresponding author.

## Supplementary Materials

The following supporting information can be downloaded at: https://www.mdpi.com/article/10.3390/polym17111515/s1, Table S1: Complete blood count data from collected blood; Table S2: Complete blood count data from isolated PRP.
