# Supplementary material for: Impact of BMI and PRP Platelet and Red Blood Cell Content on the Coagulation Kinetics of Ortho-R/PRP Mixtures
Source: Polymers (Basel). 2025 May 29;17(11):1515. doi: 10.3390/polym17111515 (PMC12157280; doi:10.3390/polym17111515)
Supplement: Supplementary file 1 [file polymers-17-01515-s001.zip › polymers-3648064-supplementary.pdf]

Table S1. Complete blood count data from collected blood.

Parameter	WBC ($X10^9/L$)	LY (%)	MO (%)	NE (%)	EO (%)	BA (%)
**All donors (n=60)**	6.4 ± 1.8	33.4 ± 8.5	7.8 ± 2.1	55.7 ± 9.8	2.9 ± 2.2	0.3 ± 0.2
**Males (n=27)**	6.1 ± 1.8	32.9 ± 8.9	8.2 ± 1.9	55.5 ± 9.7	3.2 ± 2.7	0.3 ± 0.1
**Females (n=33)**	6.6 ± 1.8	33.7 ± 8.2	7.4 ± 2.2	55.9 ± 10.0	2.7 ± 1.8	0.4 ± 0.2

WBC: White blood cells; LY: Lymphocytes; MO: Monocytes; NE: Neutrophils; EO: Eosinophils; BA: Basophils.

Parameter	LY ($X10^9/L$)	MO ($X10^9/L$)	NE ($X10^9/L$)	EO ($X10^9/L$)	BA ($X10^9/L$)	Platelets ($X10^9/L$)
**All donors (n=60)**	2.1 ± 0.7	0.5 ± 0.2	3.6 ± 1.4	0.2 ± 0.1	0.02 ± 0.01	253 ± 74
**Males (n=27)**	1.9 ± 0.5	0.5 ± 0.2	3.5 ± 1.5	0.2 ± 0.2	0.02 ± 0.01	219 ± 45
**Females (n=33)**	2.2 ± 0.8	0.5 ± 0.2	3.7 ± 1.4	0.2 ± 0.1	0.02 ± 0.01	281 ± 82

LY: Lymphocytes; MO: Monocytes; NE: Neutrophils; EO: Eosinophils; BA: Basophils.

Parameter	RBC ($X10^{12}/L$)	HB (g/L)	HT (L/L)	MCV (fl)	MCH (pg)	MCHC (g/L)
**All donors (n=60)**	4.8 ± 0.4	146.2 ± 12.9	0.43 ± 0.03	89.0 ± 4.3	30.6 ± 1.8	343.7 ± 7.7
**Males (n=27)**	5.1 ± 0.3	157.4 ± 6.3	0.45 ± 0.02	89.7 ± 3.0	31.1 ± 1.2	346.1 ± 5.6
**Females (n=33)**	4.5 ± 0.3	137.1 ± 9.0	0.40 ± 0.02	88.5 ± 5.2	30.3 ± 2.2	341.8 ± 8.6

RBC: Red blood cells; HB: Hemoglobin; HT: Hematocrit; MCV: Mean corpuscular volume; MCH: Mean corpuscular hemoglobin; MCHC: Mean corpuscular hemoglobin concentration.

Table S2. Complete blood count data from isolated PRP.

Parameter	WBC ($X10^9/L$)	LY (%)	MO (%)	NE (%)	EO (%)	BA (%)
**All donors (n=60)**	23.4 ± 9.5	60.2 ± 13.4	17.3 ± 4.7	21.6 ± 14.5	0.4 ± 0.8	0.4 ± 0.2
**Males (n=27)**	22.5 ± 8.3	56.2 ± 13.7	17.7 ± 4.6	25.1 ± 15.3	0.5 ± 1.1	0.4 ± 0.2
**Females (n=33)**	24.2 ± 10.4	63.5 ± 12.4	17.0 ± 5.0	18.8 ± 13.3	0.3 ± 0.3	0.5 ± 0.2

WBC: White blood cells; LY: Lymphocytes; MO: Monocytes; NE: Neutrophils; EO: Eosinophils; BA: Basophils.

Parameter	LY ($X10^9/L$)	MO ($X10^9/L$)	NE ($X10^9/L$)	EO ($X10^9/L$)	BA ($X10^9/L$)	Platelets ($X10^9/L$)
**All donors (n=60)**	13.8 ± 6.1	4.0 ± 1.9	5.4 ± 4.7	0.09 ± 0.14	0.11 ± 0.08	1451 ± 553
**Males (n=27)**	12.3 ± 4.7	3.9 1.5	6.1 ± 5.1	0.11 ± 0.19	0.11 ± 0.08	1339 ± 435
**Females (n=33)**	15.0 ± 6.8	4.1 ± 2.2	4.9 ± 4.3	0.07 ± 0.09	0.12 ± 0.09	1542 ± 625

LY: Lymphocytes; MO: Monocytes; NE: Neutrophils; EO: Eosinophils; BA: Basophils.

Parameter	RBC ($X10^{12}/L$)	HB (g/L)	HT (L/L)	MCV (fl)	MCH (pg)	MCHC (g/L)
**All donors (n=60)**	1.4 ± 1.1	40.8 ± 32.4	0.13 ± 0.10	95.7 ± 5.7	31.0 ± 2.0	323.4 ± 8.6
**Males (n=27)**	1.2 ± 1.0	38.0 ± 29.8	0.12 ± 0.09	96.2 ± 4.2	31.4 ± 1.4	326.2 ± 7.0
**Females (n=33)**	1.4 ± 1.2	43.1 ± 34.7	0.13 ± 0.11	95.3 ± 6.7	30.6 ± 2.3	321.1 ± 9.3

RBC: Red blood cells; HB: Hemoglobin; HT: Hematocrit; MCV: Mean corpuscular volume; MCH: Mean corpuscular hemoglobin; MCHC: Mean corpuscular hemoglobin concentration.
